# Supplementary material for: Resistance trend in bacteria isolated from corneal ulcers: A retrospective analysis from Pakistan
Source: PLoS One. 2025 Jun 5;20(6):e0325157. doi: 10.1371/journal.pone.0325157 (PMC12140391; doi:10.1371/journal.pone.0325157)
Supplement: S1 Table — (DOCX) [file pone.0325157.s001.docx]

**Supplementary Information**

**S1 Table. Antibiotic resistant spectra of bacterial isolates from corneal ulcer samples**

| **AB** | **CNS** | **MR-CoNS** | **STC** | **MRSA5** | **EC6** | **EC7** | **STM** | **PA9** | **P10** | **MRSA11** | **PA12** | **P13** | **PA14** | **HAE** | **P16** |
| --- | --- | --- | --- | --- | --- | --- | --- | --- | --- | --- | --- | --- | --- | --- | --- |
| Amk (30 μg) | 1 | 1 | 0 | 0 | 1 | 1 | 0 | 3 | 1 | 0 | 1 | 1 | 1 | 0 | 1 |
| Ceft (30 μg) | 1 | 3 | 3 | 0 | 3 | 1 | 0 | 0 | 0 | 0 | 0 | 0 | 0 | 0 | 0 |
| Clo (5 μg) | 1 | 3 | 0 | 3 | 0 | 0 | 0 | 0 | 0 | 3 | 0 | 0 | 0 | 0 | 0 |
| CoT (1.25/23.75) | 1 | 3 | 0 | 1 | 3 | 1 | 3 | 0 | 0 | 3 | 0 | 0 | 0 | 1 | 0 |
| Dox (10 μg) | 1 | 0 | 0 | 1 | 1 | 1 | 0 | 0 | 0 | 1 | 0 | 0 | 0 | 1 | 0 |
| Imp (10 μg) | 1 | 3 | 1 | 0 | 1 | 1 | 0 | 1 | 3 | 3 | 1 | 2 | 1 | 0 | 1 |
| Lnz (30 μg) | 1 | 1 | 1 | 1 | 0 | 0 | 0 | 0 | 0 | 0 | 0 | 0 | 0 | 0 | 0 |
| Min (30 μg) | 1 | 1 | 0 | 0 | 1 | 1 | 1 | 0 | 0 | 0 | 0 | 0 | 0 | 0 | 0 |
| Pen (10 μg) | 3 | 0 | 3 | 3 | 0 | 0 | 0 | 0 | 0 | 3 | 0 | 0 | 0 | 0 | 0 |
| Gen (10 μg) | 1 | 1 | 1 | 3 | 3 | 1 | 0 | 3 | 1 | 3 | 1 | 1 | 1 | 0 | 3 |
| Cep (30 μg) | 0 | 3 | 0 | 3 | 0 | 0 | 0 | 0 | 0 | 3 | 0 | 0 | 0 | 0 | 0 |
| Aug (20/10 µg) | 0 | 3 | 3 | 0 | 0 | 1 | 0 | 0 | 0 | 3 | 0 | 0 | 0 | 1 | 0 |
| Amp (10 μg) | 0 | 3 | 0 | 0 | 3 | 1 | 0 | 0 | 0 | 0 | 0 | 0 | 0 | 1 | 0 |
| Cip (5 μg) | 0 | 1 | 3 | 3 | 3 | 1 | 0 | 3 | 3 | 3 | 1 | 1 | 3 | 3 | 3 |
| Lev (5 μg) | 0 | 0 | 3 | 0 | 0 | 0 | 3 | 0 | 0 | 0 | 0 | 0 | 0 | 0 | 0 |
| Cef (30 μg) | 0 | 0 | 3 | 0 | 0 | 1 | 1 | 0 | 1 | 0 | 1 | 3 | 1 | 0 | 1 |
| Chl (30 μg) | 0 | 0 | 1 | 1 | 0 | 0 | 1 | 0 | 0 | 1 | 0 | 0 | 0 | 0 | 0 |
| Mer (31 μg) | 0 | 0 | 0 | 0 | 1 | 1 | 0 | 0 | 3 | 3 | 1 | 2 | 1 | 0 | 1 |
| Tig (15 μg) | 0 | 0 | 0 | 0 | 1 | 0 | 0 | 0 | 0 | 0 | 3 | 0 | 0 | 0 | 0 |
| Ce (30 μg) | 0 | 0 | 0 | 0 | 0 | 1 | 0 | 1 | 0 | 0 | 0 | 0 | 0 | 0 | 1 |
| Azt (30 μg) | 0 | 0 | 0 | 0 | 0 | 1 | 0 | 0 | 0 | 0 | 1 | 0 | 1 | 0 | 3 |
| Van | 1 | 1 | 1 | 1 | 0 | 0 | 0 | 0 | 0 | 1 | 0 | 0 | 0 | 1 | 0 |
| PB | 0 | 0 | 0 | 0 | 1 | 1 | 0 | 0 | 1 | 0 | 1 | 3 | 1 | 0 | 3 |
| Col | 0 | 0 | 0 | 0 | 0 | 0 | 0 | 1 | 0 | 0 | 1 | 3 | 1 | 0 | 3 |
