# Supplementary material for: Resistance trend in bacteria isolated from corneal ulcers: A retrospective analysis from Pakistan
Source: PLoS One. 2025 Jun 5;20(6):e0325157. doi: 10.1371/journal.pone.0325157 (PMC12140391; doi:10.1371/journal.pone.0325157)
Supplement: S2 Table — (DOCX) [file pone.0325157.s002.docx]

**S2 Table. Biochemical characters of the bacterial isolates obtained from corneal ulcer samples**

|  | **Culture Organism** | **ONPG** | **GLU** | **ARA** | **LDC** | **ODC** | **CIT** | **H2S** | **URE** | **TDA** | **IND** | **OX** | **NO2** | **Catalase** | **DNase** | **Coagulase** |
| --- | --- | --- | --- | --- | --- | --- | --- | --- | --- | --- | --- | --- | --- | --- | --- | --- |
| **EW1** | **CNS** | 0 | 0 | 0 | 0 | 0 | 0 | 0 | 0 | 0 | 0 | 0 | 0 | 1 | 1 | 1 |
| **EW2** | **MR-CoNS** | 0 | 0 | 0 | 0 | 0 | 0 | 0 | 0 | 0 | 0 | 0 | 0 | 1 | 1 | 1 |
| **EW3** | **STS** | 0 | 1 | 1 | 0 | 0 | 0 | 0 | 0 | 0 | 0 | 1 | 0 | 0 | 0 | 0 |
| **EW4** | **MRSA5** | 0 | 0 | 0 | 0 | 0 | 0 | 0 | 0 | 0 | 0 | 0 | 0 | 1 | 1 | 1 |
| **EW5** | **EC6** | 1 | 1 | 0 | 0 | 1 | 0 | 0 | 0 | 0 | 1 | 0 | 1 | 0 | 0 | 0 |
| **EW6** | **EC7** | 1 | 1 | 0 | 0 | 1 | 0 | 0 | 0 | 0 | 1 | 0 | 1 | 0 | 0 | 0 |
| **EW7** | **STM** | 0 | 1 | 0 | 0 | 0 | 1 | 0 | 0 | 1 | 0 | 0 | 1 | 1 | 1 | 1 |
| **EW8** | **PA9** | 0 | 0 | 0 | 0 | 0 | 0 | 0 | 0 | 0 | 0 | 1 | 0 | 0 | 0 | 0 |
| **EW9** | **P10** | 0 | 0 | 0 | 0 | 0 | 0 | 0 | 0 | 0 | 0 | 1 | 0 | 0 | 0 | 0 |
| **EW10** | **MRSA11** | 0 | 0 | 0 | 0 | 0 | 0 | 0 | 0 | 0 | 0 | 0 | 0 | 1 | 1 | 1 |
| **EW11** | **PA12** | 0 | 0 | 0 | 0 | 0 | 0 | 0 | 0 | 0 | 0 | 1 | 0 | 0 | 0 | 0 |
| **EW12** | **P13** | 0 | 0 | 0 | 0 | 0 | 0 | 0 | 0 | 0 | 0 | 1 | 0 | 0 | 0 | 0 |
| **EW13** | **PA14** | 0 | 0 | 0 | 0 | 0 | 0 | 0 | 0 | 0 | 0 | 1 | 0 | 0 | 0 | 0 |
| **EW14** | **HAE** | 0 | 0 | 0 | 0 | 0 | 0 | 1 | 1 | 0 | 1 | 1 | 1 | 0 | 0 | 0 |
| **EW15** | **P16** | 0 | 0 | 0 | 0 | 0 | 0 | 0 | 0 | 0 | 0 | 1 | 0 | 0 | 0 | 0 |
