# Supplementary material for: Resistance trend in bacteria isolated from corneal ulcers: A retrospective analysis from Pakistan
Source: PLoS One. 2025 Jun 5;20(6):e0325157. doi: 10.1371/journal.pone.0325157 (PMC12140391; doi:10.1371/journal.pone.0325157)
Supplement: Attachment — Submitted filename: Comments_Eye.docx [file pone.0325157.s004.docx]

**Response to Reviewers’ Comments**

The authors are thankful to the reviewers for their valuable insights and constructive feedback which has significantly contributed to enhance the quality of the manuscript. All the revisions are highlighted in yellow.

**Reviewer # 1**

**General comment**

The article presents significant findings on antibiotic resistance patterns from corneal ulcers. Globally, antibiotic resistance is recognized as a major public health threat. These infections can lead to visual impairment and even blindness if not treated. Pseudomonas aeruginosa shows high resistance to commonly used antibiotics such as ciprofloxacin and gentamicin, which are frequently prescribed for corneal infections. This trend is alarming, necessitating that the protocols may need to be reevaluated to manage these infections. In the context of Pakistan, the study's implications are particularly pronounced. The country faces a significant burden of infectious diseases, compounded by inadequate healthcare infrastructure and the overuse of antibiotics. Even though the sample size is small, I have not seen such a study reported in the Pakistani population. There is a lack of data on antibiotic resistance patterns in Pakistan concerning corneal ulcers. The current study fills this gap by providing valuable insights. The appealing visual illustrations of resistance patterns enhance the understanding of the relationships between different antibiotics and bacterial strains. This study not only informs clinical practice but also underscores the urgent need for public health initiatives to address antibiotic misuse.

Overall, I suggest minor revisions to improve the quality of the manuscript further.

**Comment 1**

Explain antibiotics use and the prevalence of resistance in Pakistan, especially in ophthalmic infections?

**Response**

The required information regarding **antibiotics use and the prevalence of resistance in Pakistan, particularly in ophthalmic infections,** has been added in **lines 78-80.**

**Comment 2**

Highlight how local practices may contribute to resistance patterns?

**Response**

The required information on how local practices contribute to resistance patterns has been incorporated from line 76. This section now includes details on **antibiotic overuse, self-medication, and other key factors influencing resistance trends.**

**Comment 3**

Please elaborate on the disc diffusion method. Discuss how the results were interpreted, including the criteria for determining resistance and susceptibility. This is crucial for readers to understand the significance of your findings.

**Response 3**

The disc diffusion method, also known as the Kirby-Bauer method, is a widely used technique for assessing the antibiotic susceptibility of bacterial isolates. This method follows the guidelines set by the Clinical and Laboratory Standards Institute (CLSI). The description is added from line 126.

**Comment 4**

It is important to acknowledge the role of non-bacterial agents in corneal infections. Discuss the prevalence of fungal pathogens such as Aspergillus and Candida, as well as viral and parasitic causes.

**Response**

The introduction has been expanded from line 61 to include a discussion on the prevalence of fungal pathogens, such as Aspergillus and Candida, as well as viral and parasitic causes of corneal infections. Relevant sources have been cited to highlight the significance of these pathogens in corneal infections.

**Reviewer # 2**

**General comment**

Highlight how the resistance patterns observed in the current study reflect or diverge from global trends.

The study's measurement of healing rates in corneal ulcer patients, alongside assessing antibiotic resistance levels across various bacterial strains and their specific treatment responses, is highly relevant in the context of rising antibiotic resistance. Understanding the healing rates allows for the evaluation of treatment efficacy while analysing resistance patterns, which provides critical insights into the challenges faced in managing corneal infections as well as informs more personalized treatment plans towards optimizing patient outcomes. However, here are some comments and recommendations.

**Comment 1**

The timeline of the study should be reviewed and consistently stated throughout the document to ensure clarity.

**Response**

The timeline has been carefully reviewed and ensure consistency throughout the document to improve clarity.

**Comment 2**

The sample size utilized in this study is insufficient.

**Response**

The incidence of corneal infections is relatively low, and patient recruitment was inherently limited by case availability during the study period. Notably, corneal ulcer cases are more prevalent during the summer and monsoon seasons, which may be attributed to increased exposure to environmental pathogens and humid conditions favouring microbial growth. Nevertheless, the findings provide valuable insights into corneal ulcer pathology and microbial patterns. Future studies with larger cohorts will further substantiate these observations.

**Comment 3**

Currently, relying solely on disc diffusion test results is not widely accepted for detecting antibiotic resistance. Therefore, it is recommended that the authors validate their disc diffusion results, particularly for resistant strains, using additional approved methods such as the microdilution method or E-test. Employing these techniques will provide accurate minimum inhibitory concentration (MIC) values for each resistant antibiotic strain identified.

**Response**

Due to economic constraints in Pakistan, the disc diffusion method remains the most widely used and feasible approach in clinical settings. Despite its limitations, it provides reliable and reproducible results under local laboratory conditions. In the current study, E-test is typically performed for vancomycin-resistant strains; however, as no vancomycin resistance was observed, further MIC determination was not required.

**Comment 4**

The detection of vancomycin resistance through disc diffusion is not considered valid according to CLSI and EUCAST guidelines. It is essential to perform E-tests alongside microdilution methods to assess vancomycin resistance and investigate the presence of VanA, VanB, and VanC genes in the isolated strains.

**Response**

No vancomycin-resistant strains were identified in this study. The authors acknowledges that as per CLSI and EUCAST guidelines, disc diffusion alone is not a reliable method for detecting vancomycin resistance. However, all isolates showed susceptibility to vancomycin based on the disc diffusion method, and no resistance patterns were observed that would justify further confirmation by E-test, broth microdilution, or PCR for VanA, VanB, and VanC genes. Future studies will consider molecular confirmation where necessary.

**Comment 5**

Although phenotypic approaches are valuable, integrating high-throughput molecular methods would yield data, leading to stronger conclusions. Notwithstanding, the authors did impressive work regarding their statistical analysis. The highlights of some of the statistical analysis, especially from the frequency of interactions between each antibiotic and bacterial strain, can be stated in the abstract.

**Response**

Abstract has been modified accordingly.

I hope that the responses have satisfied the queries/ comments of the reviewers. Once again I am thankful to the reviewers whose input has raised the quality of the manuscript.

**Dr. Sidra Abbas**
