# Supplementary material for: Resistance trend in bacteria isolated from corneal ulcers: A retrospective analysis from Pakistan
Source: PLoS One. 2025 Jun 5;20(6):e0325157. doi: 10.1371/journal.pone.0325157 (PMC12140391; doi:10.1371/journal.pone.0325157)
Supplement: Attachment — Submitted filename: Reviewe Comments 2nd R.docx [file pone.0325157.s005.docx]

The study addresses a critical public health issue of antibiotic resistance in corneal ulcer pathogens in Pakistani region with limited data. The topic is timely and clinically relevant as I stated in my previous review comments also. But I have noticed many discrepancies in the revised manuscript which are as following;

In response to the comment “Explain antibiotics use and the prevalence of resistance in Pakistan, especially in ophthalmic infections”, The authors addressed the comment partially which is not satisfactory. The authors acknowledged empirical antibiotic use (fluoroquinolones, aminoglycosides) and drivers of resistance (self-medication, over the counter availability of drugs). Quantitative data on resistance prevalence, such as national and regional resistance from Pakistan, is missing. The text lacks data and citations on local ophthalmic infections related antibiotic resistance.

The authors response to address the comment “Please elaborate on the disc diffusion method. Discuss how the results were interpreted, including the criteria for determining resistance and susceptibility. This is crucial for readers to understand the significance of your findings.” is incomplete. The CLSI reference is noted, but critical details are missing. Specific CLSI breakpoints for each antibiotic have not been enlisted. No quality control strains have been used to validate the method.

I cannot find the addressal of the comment “Highlight how the resistance patterns observed in the current study reflect or diverge from global trends.” In the discussion section of the article. The authors fail to compare findings to regional studies. For example, there is no mention of P. aeruginosa resistance rates to ciprofloxacin in South Asia/Pakistan/India and global data. This comment has not been addressed.

The authors need to justify why fungal/viral testing was not performed despite Sabouraud agar use to confirm any co-infection in patients. Discuss the implications of missing fungal/viral co-infection data.

Kirby-Bauer disc diffusion alone for vancomycin susceptibility is invalid as per CLSI guidelines. Disc diffusion lacks reliability for glycopeptides and carbapenems.

Biochemical tests for bacterial identification lack specificity. Biochemical characterization is rudimentary. Misidentification of *Staphylococcus aureus* , like coagulase-negative isolates labeled as MRSA raises concerns. No secondary confirmation test was performed, such as broth microdilution and molecular assays.

It is better to confirm species using 16S rRNA sequencing.

Mixed infections are common in corneal ulcers and influence resistance profiles. Perform fungal cultures or PCR and viral testing to rule out co-infections.

The retrospective design lacks a control group, reducing the ability to infer causality or resistance trends.

The choice of 26 antibiotics is broad but lacks justification. Why Nitrofurantoin antibiotic also checked as it is used in urinary tract infections?

The conclusion is generic and lacks specificity. It overstates the study’s impact given the small sample size.

**S**ome citations (Woodland et al., 1992, Title: Causes of conjunctivitis and keratoconjunctivitis in Karachi, Pakistan) are outdated. Include recent studies.

The authors did not mention inclusion and exclusion criteria.

**Decision**

**Major Revisions Suggested.** Most of the comments have been addressed superficially. The manuscript suffers from inconsistencies.
