# Supplementary material for: Resistance trend in bacteria isolated from corneal ulcers: A retrospective analysis from Pakistan
Source: PLoS One. 2025 Jun 5;20(6):e0325157. doi: 10.1371/journal.pone.0325157 (PMC12140391; doi:10.1371/journal.pone.0325157)
Supplement: Attachment — Submitted filename: Eye_C2_18April.docx [file pone.0325157.s006.docx]

**Response to Reviewers’ Comments**

The authors are thankful to the reviewers for their valuable insights and constructive feedback which has significantly contributed to enhance the quality of the manuscript. All the revisions are highlighted in yellow.

**Reviewer Comments**

The study addresses a critical public health issue of antibiotic resistance in corneal ulcer pathogens in Pakistani region with limited data. The topic is timely and clinically relevant as I stated in my previous review comments also. But I have noticed many discrepancies in the revised manuscript which are as following.

**Comment 1**

In response to the comment “Explain antibiotics use and the prevalence of resistance in Pakistan, especially in ophthalmic infections”, The authors addressed the comment partially which is not satisfactory. The authors acknowledged empirical antibiotic use (fluoroquinolones, aminoglycosides) and drivers of resistance (self-medication, over the counter availability of drugs). Quantitative data on resistance prevalence, such as national and regional resistance from Pakistan, is missing. The text lacks data and citations on local ophthalmic infections related antibiotic resistance.

**Response**

Quantitative data on resistance prevalence at the regional level have been added in the introduction page 3.

**Comment 2**

The authors response to address the comment “Please elaborate on the disc diffusion method. Discuss how the results were interpreted, including the criteria for determining resistance and susceptibility. This is crucial for readers to understand the significance of your findings.” is incomplete. The CLSI reference is noted, but critical details are missing. Specific CLSI breakpoints for each antibiotic have not been enlisted. No quality control strains have been used to validate the method.

**Response**

The disc diffusion method has been revised on page 5. Specific CLSI breakpoints for each antibiotic have been included results and discussion in the under the heading **antibiotic resistance profile of bacterial species.** However, in clinical pathology laboratory, antibiotic susceptibility testing for different samples from different departments in the hospitals including the corneal ulcers are performed as part of routine diagnostic workflow, where hundreds of samples are processed daily under standardized operating procedures. While ideal for research studies, running concurrent QC strains with every routine clinical specimen is not feasible due to:

Operational Considerations:

As a high-volume diagnostic laboratory processing hundreds of specimens daily, we prioritize, timely reporting for patient care, Batch-level QC validation, Strict adherence to standardized operating procedures

Standardized Identification Methods:

All isolates underwent biochemical confirmation using API test kits (bioMérieux), consistent with established methodologies given below as well (Khor et al., 2024; Naik et al., 2021).

**Comment 3**

I cannot find the addressal of the comment “Highlight how the resistance patterns observed in the current study reflect or diverge from global trends.” In the discussion section of the article. The authors fail to compare findings to regional studies. For example, there is no mention of P. aeruginosa resistance rates to ciprofloxacin in South Asia/Pakistan/India and global data. This comment has not been addressed.

**Response**

Resistance patterns of the tested antibiotics to regional/global data, emphasizing critical disparities have been added in the text on page 10 and 11.

**Comment 4**

The authors need to justify why fungal/viral testing was not performed despite Sabouraud agar use to confirm any co-infection in patients. Discuss the implications of missing fungal/viral co-infection data.

**Response**

Fungal and viral keratitis cases were excluded from this study, as the focus was on admitted patients with bacterial keratitis. As part of our standard microbiological protocol, corneal scrapings from all patients were cultured on Sabouraud agar to screen for potential fungal co-infections. However, patients diagnosed with fungal or viral keratitis were managed separately and not included in the final analysis to maintain a focused and uniform study population.

**Comment 5**

Kirby-Bauer disc diffusion alone for vancomycin susceptibility is invalid as per CLSI guidelines. Disc diffusion lacks reliability for glycopeptides and carbapenems.

**Response**

Broth microdilution (BMD) MIC testing has now been incorporated for vancomycin susceptibility, as required by CLSI guidelines for glycopeptides. The revised manuscript includes these updated methods in the Methodology section.

**Comment 6**

Biochemical tests for bacterial identification lack specificity. Biochemical characterization is rudimentary. Misidentification of *Staphylococcus aureus* , like coagulase-negative isolates labeled as MRSA raises concerns. No secondary confirmation test was performed, such as broth microdilution and molecular assays.

**Response**

We acknowledge that referring to coagulase-negative isolates as MRSA was a typographical error. In our hospital setting, biochemical identification remains a standard and practical approach for timely diagnosis and reporting, particularly given the high volume of samples and the need to initiate prompt treatment. While we recognize the superior specificity of molecular assays such as PCR, resource limitations and financial constraints precluded their use in this study. Nonetheless, the biochemical methods employed are routinely used in clinical microbiology and are considered sufficiently reliable for initial identification and management.

**Comment 7**

It is better to confirm species using 16S rRNA sequencing.

**Response**

Thank you for the suggestion. We agree that 16S rRNA sequencing is a highly reliable and accurate method for bacterial species identification. However, due to the high volume of samples and limited financial and laboratory resources, it was not feasible to incorporate molecular sequencing methods in our study. Our identification protocol relied on conventional biochemical testing, which is routinely used in clinical microbiology laboratories for timely and cost-effective diagnosis. While we acknowledge that 16S rRNA sequencing would enhance the precision of species confirmation, the focus of our study was on clinical outcomes and management, for which conventional identification methods were considered sufficient within the given constraints. Below are the few studies which used biochemical and the sensitivity assays for the identification of the bacterial strains.

Khor, W.B., Lakshminarayanan, R., Periayah, M.H., Prajna, V.N., Garg, P., Sharma, N., Mehta, J.S., Young, A., Goseyarakwong, P., Puangsricharern, V. and Tan, A.L., 2024. The antibiotic resistance profiles of *Pseudomonas aeruginosa* in the Asia Cornea Society Infectious Keratitis Study. *International Ophthalmology*, *44*(1), p.361.

Fernandes, M., Vira, D., Medikonda, R. and Kumar, N., 2016. Extensively and pan-drug resistant *Pseudomonas aeruginosa* keratitis: clinical features, risk factors, and outcome. *Graefe's Archive for Clinical and Experimental Ophthalmology*, *254*, pp.315-322.

Naik, P., Pandey, S., Gagan, S., Biswas, S. and Joseph, J., 2021. Virulence factors in multidrug (MDR) and Pan-drug resistant (XDR) *Pseudomonas aeruginosa*: a cross-sectional study of isolates recovered from ocular infections in a high-incidence setting in southern India. *Journal of Ophthalmic Inflammation and Infection*, *11*, pp.1-11.

Cabrera‐Aguas, M., Chidi‐Egboka, N., Kandel, H. and Watson, S.L., 2024. Antimicrobial resistance in ocular infection: A review. *Clinical & Experimental Ophthalmology*, *52*(3), pp.258-275.

AlBahrani, S., Alqazih, T.Q., Aseeri, A.A., Al Argan, R., Alkhafaji, D., Alrqyai, N.A., Alanazi, S.M., Aldakheel, D.S., Ghazwani, Q.H., Jalalah, S.S. and Alshuaibi, A.K., 2024. Pattern of cephalosporin and carbapenem-resistant *Pseudomonas aeruginosa*: a retrospective analysis. *IJID regions*, *10*, pp.31-34.

**Comment 8**

Mixed infections are common in corneal ulcers and influence resistance profiles. Perform fungal cultures or PCR and viral testing to rule out co-infections.

**Response**

Thank you for the valuable comment. For all admitted patients with corneal ulcers, corneal scrapings were collected and processed for microbiological evaluation, including staining and culture on blood agar, chocolate agar, and Sabouraud dextrose agar. Smears were also prepared for microscopic examination. Patients who showed a positive culture and sensitivity to a specific antibiotic were started on targeted therapy and included in the study. Cases of viral keratitis were excluded, as these patients were managed on an outpatient basis and not part of the admitted cohort. Similarly, patients with fungal keratitis who developed complications such as non-healing ulcers or corneal perforation were excluded from the final analysis. However, patients presenting with mixed infections involving both bacterial and fungal pathogens, who responded to treatment, were included in the study.

**Comment 9**

The retrospective design lacks a control group, reducing the ability to infer causality or resistance trends.

**Response**

Thank you for the comment. However, we respectfully disagree with the suggestion regarding the need for a control group in this context. The retrospective design of our study was intentionally chosen to allow for the efficient analysis of resistance patterns in clinically confirmed cases of infectious keratitis. The primary objective was to characterize antimicrobial resistance in real-world clinical isolates rather than to assess treatment efficacy or outcomes between different groups. Given the ethical considerations, it would not be appropriate to withhold diagnostic procedures, such as corneal scrapings, from symptomatic patients solely to establish an untreated control group. Additionally, control groups are more commonly utilized in prospective clinical trials or interventional studies, whereas retrospective observational studies, particularly those focused on microbiological profiling, do not typically include control arms. Therefore, the absence of a control group does not compromise the validity of our findings within the scope of this study's objectives.

**Comment 10**

The choice of 26 antibiotics is broad but lacks justification. Why Nitrofurantoin antibiotic also checked as it is used in urinary tract infections?

**Response**

We acknowledge the reviewer’s concern regarding the inclusion of nitrofurantoin in our antibiotic panel. This was inadvertently added due to an administrative error during table compilation and was originally intended for a separate study on urinary tract infections. We sincerely apologize for this oversight and confirm that nitrofurantoin testing was not performed in our corneal ulcer study. The updated manuscript which now reflects only clinically relevant antibiotics used for ocular infections, with detailed justifications provided for each agent based on CLSI guidelines.

**Comment 11**

The conclusion is generic and lacks specificity. It overstates the study’s impact given the small sample size.

**Response**

Conclusion have been revised

**Comment 12**

**S**ome citations (Woodland et al., 1992, Title: Causes of conjunctivitis and keratoconjunctivitis in Karachi, Pakistan) are outdated. Include recent studies.

**Response**

Recent references have been included, and the reference are updated accordingly.

**Comment 13**

The authors did not mention inclusion and exclusion criteria.

**Response**

Inclusion and exclusion criteria have now been added in the manuscript.

**Decision**

**Major Revisions Suggested.** Most of the comments have been addressed superficially. The manuscript suffers from inconsistencies.

I hope that the responses have satisfied the queries/ comments of the reviewers. Once again I am thankful to the reviewers whose input has raised the quality of the manuscript.

**Dr. Sidra Abbas**
