# Supplementary material for: Resistance trend in bacteria isolated from corneal ulcers: A retrospective analysis from Pakistan
Source: PLoS One. 2025 Jun 5;20(6):e0325157. doi: 10.1371/journal.pone.0325157 (PMC12140391; doi:10.1371/journal.pone.0325157)
Supplement: Attachment — Submitted filename: Reviewer comments and recommendation.docx [file pone.0325157.s003.docx]

The study's measurement of healing rates in corneal ulcer patients, alongside assessing antibiotic resistance levels across various bacterial strains and their specific treatment responses, is highly relevant in the context of rising antibiotic resistance. Understanding the healing rates allows for the evaluation of treatment efficacy while analyzing resistance patterns, which provides critical insights into the challenges faced in managing corneal infections as well as informs more personalized treatment plans towards optimizing patient outcomes. However, here are some comments and recommendations.

- The timeline of the study should be reviewed and consistently stated throughout the document to ensure clarity.
- The sample size utilized in this study is insufficient.
- Currently, relying solely on disc diffusion test results is not widely accepted for detecting antibiotic resistance. Therefore, it is recommended that the authors validate their disc diffusion results, particularly for resistant strains, using additional approved methods such as the microdilution method or E-test. Employing these techniques will provide accurate minimum inhibitory concentration (MIC) values for each resistant antibiotic strain identified.
- The detection of vancomycin resistance through disc diffusion is not considered valid according to CLSI and EUCAST guidelines. It is essential to perform E-tests alongside microdilution methods to assess vancomycin resistance and investigate the presence of VanA, VanB, and VanC genes in the isolated strains.
- Although phenotypic approaches are valuable, integrating high-throughput molecular methods would yield data, leading to stronger conclusions. Notwithstanding, the authors did impressive work regarding their statistical analysis. The highlights of some of the statistical analysis, especially from the frequency of interactions between each antibiotic and bacterial strain, can be stated in the abstract.
